# ASO Author Reflections: Textbook Outcomes Following Liver Resection for Hepatic Neoplasms: A Realizable and Predictable Surgical Endpoint in the Real-World Scenario

**DOI:** 10.1245/s10434-024-16102-1

**Published:** 2024-08-21

**Authors:** Kaival K. Gundavda, Shraddha Patkar, Mahesh Goel

**Affiliations:** Department of Gastrointestinal and Hepatobiliary Surgery, Department of Surgical Oncology, Tata Memorial Hospital, Homi Bhabha National Institute (HBNI), Mumbai, Maharashtra India

## Past

Liver resection remains a technically challenging surgery with potential for postoperative morbidity.^1^ Traditional outcome measures such as the Clavien–Dindo Classification (CDC) system and the Comprehensive Complication Index (CCI) are not tailored to the post-hepatectomy setting. This limitation was overcome when the International Study Group of Liver Surgery (ISGLS) proposed a classification of complications unique to hepatobiliary surgery.^2–4^ Despite this, there was an unmet need for a comprehensive and unified system to define the ideal postoperative course after liver resection and enable standardization and global comparison. Textbook Outcomes (TO) were developed to improve the quality of hospital care and prioritize patient-centered care. It was not until 2023 that an international expert consensus-based definition was proposed, using the formal Delphi approach.^5^ Regrettably, only 4.6% of the expert panel members represented lower–middle-income and low-income economies.

## Present

We recognized the need for a pragmatic model to serve as a predictive tool for surgical outcomes following liver resections in the outpatient clinic, with a focus on achieving the ideal postoperative outcomes. In this study,^6^ we assess the reliability of TO as an outcome measure in a real-world setting in a low–middle-income economy. Over 12 years and 1018 consecutive liver resections, we achieved a TO rate of 64.9% (661/1018), comparable to world literature. We discovered that TO was independently associated with improved survival. If TO embodies the “ideal postoperative outcome,” we identified predictors and developed a risk-scoring system to predict the postoperative course. Adverse predictors of TO include a diagnosis of perihilar cholangiocarcinoma, major hepatectomy (≥ 3 segment resection), intraoperative blood loss > 1500 ml, and the presence of lymphovascular emboli on pathology. By integrating essential perioperative parameters, we developed and validated a simplified nomogram for the prediction of TO after liver resection.

## Future

TO after liver resection is a realizable outcome measure and should be adopted. We recommend using the proposed nomogram as a convenient tool for patient selection and prognosticating outcomes following hepatectomy. The future may allow online calculators and artificial intelligence (AI)-based tools to help guide patient selection for surgical resection. A calculated score predicting a higher risk could allow for surgical preparedness, informed decision-making, postoperative risk stratification, and objective patient counselling. Anticipating challenges in postoperative recovery (indicated by a high TO score) could also indicate the need to refer the patient to high-volume specialized centres, especially in resource-limited settings.
